# The Influence of Methods for Distributing the IF-WS_2_ Modifier into the Structure of Al_2_O_3_ Aluminium Oxide Coatings on Their Micromechanical Properties †

**DOI:** 10.3390/ma19040667

**Published:** 2026-02-09

**Authors:** Joanna Korzekwa, Adrian Barylski, Mateusz Niedźwiedź, Krzysztof Cwynar, Marek Bara

**Affiliations:** 1 Institute of Materials Engineering, University of Silesia in Katowice, 41-500 Chorzow, Poland; mateusz.niedzwiedz@us.edu.pl (M.N.); marek.bara@us.edu.pl (M.B.); 2 Institute of Chemistry, Faculty of Science and Technology, University of Silesia in Katowice, 40-007 Katowice, Poland

**Keywords:** aluminium oxide coatings, IF-WS_2_ nano lubricant, micromechanical properties

## Abstract

This work examines the micromechanical response of Al_2_O_3_/IF-WS_2_ (IF-inorganic fullerene-like) composite coatings formed on the EN AW 5251 aluminium alloy by anodic oxidation. The resulting amorphous oxide layer contains a nanopores system that can be filled with IF-WS_2_ particles, provided the modifier is properly dispersed. Because commercial IF-WS_2_ powders exhibit strong agglomeration, a high-intensity ultrasonic treatment was applied to enhance particle separation before incorporation. The influence of newly established incorporation parameters was assessed using a two-level experimental design. As part of the research, analyses of the microstructure, micromechanical, and sclerometric properties were performed. Cross-sectional SEM observations confirmed the presence of IF-WS_2_ within the oxide structure and revealed differences in particle distribution, depending on the incorporation technique used. The results indicate that although microhardness and Young’s modulus are largely insensitive to the nanopowder incorporation method, the interaction between the anodising current density and the incorporation technique significantly influences the strain energy components and tribological response of the coatings. These findings suggest that appropriately selected processing parameters can be used to tailor the mechanical and tribological properties of Al_2_O_3_/IF-WS_2_ coatings to specific loading conditions and functional requirements, rather than striving for a single, universal, optimal processing configuration.

## 1. Introduction

High-performance aluminium alloys are widely used in engineering applications across the transportation and industrial sectors because they offer a favourable combination of low mass and structural capability. Despite these advantages, the surfaces of aluminium alloys exhibit limited resistance to friction-induced adhesive wear unless appropriately modified. As a result, numerous studies have focused on methods for forming durable anodic oxide layers that enhance the tribological behaviour of aluminium-based components [1,2]. Such honeycomb-structured matrices are significant due to their applications in nanodots, nanorods, nanowires, and nanotubes [3,4,5]. In recent decades, considerable research efforts have focused on developing various modifications to enhance the corrosion performance of anodic coatings on aluminium alloys. Information on studies of aluminium oxide coatings in tribological applications is also available, although it is significantly less abundant. Such coatings are commonly applied to improve the functional performance of metal surfaces, offering increased hardness and enhanced resistance to wear and corrosion. The porous nature of anodic oxide films also provides an opportunity to introduce functional additives into the coating, enabling the development of surfaces with lubricating capabilities. When filled, the pore network can reduce friction and wear during operation [6,7,8,9,10,11]. Historically, one of the main obstacles in such approaches has been the disparity between the characteristic dimensions of solid lubricant particles and the nanoscale pore structure of anodic alumina, which limits efficient incorporation. In addition, excessive thinning of the oxide layer was shown to degrade the surface’s mechanical integrity [12,13]. Growing interest in recent years has therefore shifted toward the use of nanosized lubricant additives and advanced methods for integrating them into anodic coatings, offering improved compatibility with the pore architecture and enhanced tribological performance [14,15,16,17,18,19,20].

The study of micromechanical and sclerometric properties is crucial for characterising anodic coatings. Micromechanical testing, including nanoindentation and scratch tests, enables the precise evaluation of hardness, elastic modulus, and adhesion strength. Sclerometric analysis provides further insights into the scratch resistance and mechanical durability of the anodic layer. Understanding these properties is essential for optimising coating performance in various industrial applications. Micromechanical and tribological dependencies on porosity have been demonstrated, among others, for coatings produced on AA1050 [21] and high-purity aluminium [22]. Fratila-Apachitei et al. [23] showed that increasing the current density from 3.0 to 6.0 A/dm^2^ during anodising for 50 min does not improve the average microhardness of the oxide layer but instead causes significant fluctuations. Extending the anodising time increases the layer thickness while reducing its average microhardness. In aluminium alloys, microhardness assessment is challenging due to macroscopic defects and entrapped silicon particles. Sulka et al. [24] observed that the average interpore distance and pore diameter values increase with the rising anodising potential. Meanwhile, the network porosity decreases as the cell potential increases. The complexity of the wear mechanisms of oxide coatings on aluminium alloys and the associated micromechanical parameters were shown in the work of a group of researchers [25,26,27,28,29]. Xia et al. [30] determined the Young’s modulus of the produced aluminium oxide to be 140 GPa. They also found that heat treatment at 650 °C increases the hardness of aluminium oxide from 5.2 to 6.3 GPa, while the presence of pores in the oxide contributes to greater transverse strength. Vojkuvka et al. [31] demonstrated that the mechanical properties of nanoporous anodised alumina samples depend on the porosity level and the type of acidic electrolyte used in the fabrication process. Gyu-Sun Lee et al. [32] observed that porous anodic alumina templates soaked in water for 30 min showed lower friction and a more extended transition to a steady state than those that were not soaked. Pores act as reservoirs for lubrication, releasing water during elastic–plastic deformation. However, the tribochemical oxide layer has a more significant effect than the lubricant reservoir. Abedini and Hanke [33] optimised the pore widening of the anodic coating in phosphoric acid to achieve the desired properties, improving the adhesion of the nickel layer to the coating and investigating its wear resistance. Gorokh et al. [34] confirmed in their studies that the microhardness of oxide coatings increased as the pore diameter decreased. In contrast, the coating’s indentation depth increased with the size of the carbon nanostructures used for doping. This demonstrated the coatings’ excellent tribological and mechanical properties, as well as their high practical value. In our study, we addressed the challenge of nanoparticle agglomeration, which is inherently linked to the nanopowder quality [19]. In one of our earlier studies, we reported the results of an experiment aimed at enhancing the dispersion of inorganic, fullerene-like tungsten disulfide (IF-WS_2_) through high-intensity ultrasonication, combined with newly developed conditions that facilitate the introduction of the powder into the nanopores of Al_2_O_3_ coatings [35]. Due to the complex nature of nanoparticle-modified anodic layer formation and the fact that both the process of introducing additives and their distribution within the coating structure can significantly affect performance, detailed studies are necessary to examine their microstructure and behaviour under mechanical loading. Parameters such as microhardness, modulus of elasticity, contribution of plastic and elastic deformation, and scratch resistance allow for us to assess whether modification with IF-WS_2_ powder actually translates into beneficial changes in the layer’s performance, especially under tribological conditions. Equally important is determining the extent to which the nanoparticle introduction method and anodising conditions affect the coating’s mechanical stability, i.e., conducting observations under critical loads. This approach, combined with the DOE design presented in this article, represents a significant step towards the design of modified anodic oxide layers with improved functional properties, especially for applications requiring mechanical stability and high tribological resistance.

## 2. Materials and Methods

### 2.1. Sample Preparation

The substrate selected for this study was the EN AW-5251 aluminium alloy. This alloy belongs to the 5xxx series, where magnesium is the principal alloying element, providing an excellent combination of moderate strength and high corrosion resistance, particularly in marine and atmospheric environments. Additionally, EN AW-5251 offers good weldability, formability, and surface finish, making it particularly suitable for sheet forming and anodising applications. These features justify its use in applications requiring lightweight, corrosion-resistant components, such as in the automotive, aerospace, and construction industries. The Al_2_O_3_/IF-WS_2_ composite coating investigated in this study was fabricated using a two-stage preparation route. In the first stage, a graded aluminium oxide layer was generated on EN AW-5251 (Table 1) alloy by electrochemical oxidation in a ternary acid electrolyte composed of sulfuric, oxalic, and phthalic acids (Avantor Performance Materials Poland S.A., Gliwice, Poland). Prior to anodising, the specimens were subjected to a sequential chemical pretreatment involving immersion in KOH and, subsequently, in HNO_3_ solutions (both supplied by Avantor Performance Materials Poland S.A., Gliwice, Poland). Hard anodising was then conducted under a total charge of 180 A·min/dm^2^ (Table 2), with the current density, processing time, and bath temperature maintained at 303 ± 1 K. The thickness of the Al_2_O_3_ coatings produced under the experimental conditions is 44.19 ± 2.11 μm and 45.82 ± 1.40 μm for 3 A/dm^2^ and 6 A/dm^2^, respectively.

During the second stage of processing, IF-WS_2_ nanoparticles (NanoMaterials Ltd., Yavne, Israel) were introduced into the pore structure of the anodic alumina, using two alternative incorporation techniques. In method 1, the anodised samples were submerged in an ethanol suspension containing 15 g/L of IF-WS_2_ nanoparticles, treated with 20 kHz ultrasonication for 5 min, delivering 10 kJ of energy, and subsequently left to settle for 24 h. In method 2, after the initial ultrasonic dispersion step, the samples were further treated with a 15 min ultrasonic bath before being removed from the suspension [35].

### 2.2. Microstructural Studies

To investigate the composition of nanosized Al_2_O_3_ fibres incorporating self-organised IF-WS_2_ nanoparticles (NPs), the prepared samples were analysed using a HITACHI S-4700 scanning electron microscope (SEM) (Hitachi High-Tech Corporation, Tokyo, Japan) equipped with a NORAN Vantage digital energy-dispersive X-ray spectroscopy (EDS) system (NORAN Instruments, Inc., Middleton, WI, USA). The surface and cross-sectional morphology of the Al_2_O_3_/IF-WS_2_ NP coatings were examined in secondary electron (BSE) mode, utilising an yttrium aluminium garnet (YAG) detector (Crytur, Turnov, Czech Republic). A YAG single crystal was used to minimise the electron dose. The cross-sectional analysis of the Al_2_O_3_/IF-WS_2_ NPs was conducted on freshly fractured samples. Before imaging, a thin carbon film was sputter-coated onto the specimens to enhance contrast and mitigate the image distortion caused by charge accumulation.

### 2.3. Micromechanical Properties Methodology

Micromechanical tests were performed using a Micro Combi Tester—MCT3 (Anton Paar, Neuchâtel, Switzerland). A Vickers diamond indenter (V-M 86) (Vickers Ltd., London, UK) was used, with a maximum load of 500 mN (50.99 gF). Loading and unloading were conducted over 30 s (at a rate of 1000 mN/min), with a 10 s holding time at peak load. Each sample underwent eight indentations, with a spacing of 150 µm in both the x and y axes, as specified by ISO 14577 [36]. Microhardness (*H_IT_*) and elastic modulus (*E_IT_*) were determined using the Oliver–Pharr method [37]. From the recorded load–unload curves, the total indentation work (*W_total_*) and its components—plastic deformation work (*W_plast_*) and elastic deformation work (*W_elast_*)—were calculated. When evaluating Vickers hardness, it is effective to use the indentation work by plastic deformation, *W_plast_* [38]. The percentage of elastic recovery work (*η_IT_*) and the maximum depth of indenter insertion (*h_max_*) were also determined.

### 2.4. Methodology of Sclerometric Testing

The scratch resistance of the investigated layers was assessed using a Micro Combi Tester—MCT3 (Anton Paar, Neuchâtel, Switzerland), following the scratch-test method. The testing procedures adhered to the guidelines of ISO 19252 [39], ISO 20502 [40], ASTM C1624 [41], and ASTM D7027 [42], employing a Rockwell diamond indenter with a 200 μm tip diameter. Each test consisted of three stages: pre-scan—the sample profile was scanned under a 0.03 N load; scratch test (scan)—the primary test was performed with a progressively increasing load from 0.03 to 20 N; and post-scan—the scratch profile was rescanned under a 0.03 N load to evaluate the surface deformation. Each scratch was 6 mm long, with an indenter movement speed of 12 mm/min.

During the tests, the following parameters were recorded: normal force (*Fn*) [N], friction force (*Ft*) [N], indenter penetration depth under load (*Pd*) [μm], residual penetration depth after unloading (*Rd*) [μm], and acoustic emission (*AE*) [%]. Three critical loads (*L_C_*) were identified: *L_C__1_*—the onset of arc tensile cracks (Hertzian cracks) within the scratch track, often accompanied by circular Hertz tensile cracks; *L_C__2_*—the first occurrence of V-shaped forward-directed cracks at the inner and outer edges of the scratch track; and *L_C__3_*—complete coating failure, characterised by continuous perforation of the coating. The scratch test on the coating surface was conducted in two mutually perpendicular directions to evaluate potential differences in the measured sclerometric values. This methodology enables the evaluation of adhesion strength, coating durability, and failure mechanisms under progressive mechanical loads, providing critical insights into the mechanical behaviour of the protective layers.

### 2.5. Experimental Design

A 2^k^ factorial design experiment without replication was conducted using Statistica 13 software to evaluate the influence of input parameters on the dependent variables. The independent variables were current density (3 and 6 A/dm^2^) and method (1 and 2). The dependent variables for micromechanical properties were microhardness (*H_IT_*), Young’s modulus (*E_IT_*), elastic deformation work (*W_elast_*), plastic deformation work (*W_plast_*), total indentation work (W*_total_*), and elastic recovery work (*η_IT_*), and for sclerometric testing, they were friction force (*Ft*) and *L_C__1_*, *L_C__2_*, and *L_C__3_*. To verify model assumptions, normal probability plots of residuals were used to assess whether the residuals (i.e., differences between observed and predicted values) followed a normal distribution, a crucial assumption for linear regression. If residuals are normally distributed, the data points align along a straight line; however, deviations suggest departures from normality. The statistical analysis results were also used to visualise marginal mean plots with 95% confidence intervals. The ANOVA (analysis of variance) test was used to assess differences in critical load (*L_C_*) measurements in two mutually perpendicular directions. In ANOVA, one-dimensional significance tests assess differences among group means for a single factor. These tests, such as the F-test, assess whether observed variation is due to actual differences between groups or to random fluctuations. A benchmark analysis was performed using a factorial design and a two-factor interaction model. A Pareto chart was employed to identify the most influential factors, as it effectively ranks effects by magnitude and statistical significance. In statistical analysis, the *p*-value is commonly used to assess the importance of a factor. In the Pareto chart, bars crossing the reference line (*p* = 0.05) indicate statistical significance at 0.05. A detailed discussion of the DOE methodology can be found in studies [43,44,45].

## 3. Results and Discussion

To modify the functional behaviour of the anodised Al_2_O_3_ layer, inorganic fullerene-like tungsten disulfide (IF-WS_2_) nanoparticles were incorporated into its nanoporous structure. IF-WS_2_ is characterised by a hollow, multilayered spherical morphology [46], which provides advantageous mechanical responses under local loading. Owing to their layered architecture and ability to undergo shear between concentric WS_2_ shells, these nanoparticles can facilitate stress accommodation within the oxide matrix, leading to more favourable deformation behaviour [47].

**Figure 1:**
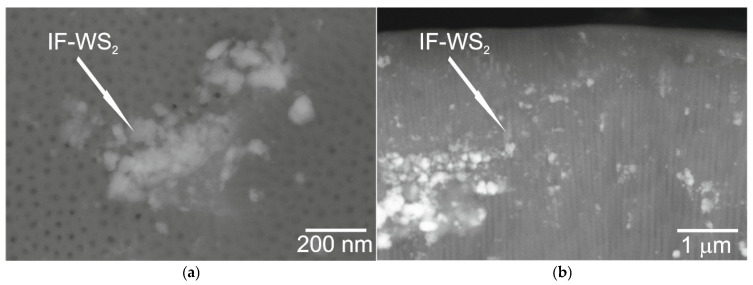
Microstructures of the surface (**a**,**b**) fresh cross-section of the Al_2_O_3_/IF-WS_2_ coating no. 1 [48].

Their thermal and chemical stability further supports their use as robust nanomodifiers that are capable of operating under demanding conditions. In the context of anodic oxide films, achieving uniform dispersion of IF-WS_2_ is essential, as agglomeration can alter the local micromechanical response and affect parameters such as indentation work and scratch resistance. Therefore, special emphasis was placed on improving nanoparticle dispersion within the Al_2_O_3_ coating to ensure consistent mechanical performance. Figure 1a,b show examples of the SEM/YAG-BSE images of the surface and cross-sections of Al_2_O_3_/IF-WS_2_ coatings from sample no. 1. The photos highlight the embedded IF-WS_2_ in coatings. The use of method 1 allowed for a more homogeneous introduction of IF-WS_2_ nanoparticles into the structure of Al_2_O_3_ nanofibers, although, as shown in Figure 1, agglomeration of the nanolubricant still occurs. The microstructure is described in more detail in [35].

**Figure 2:**
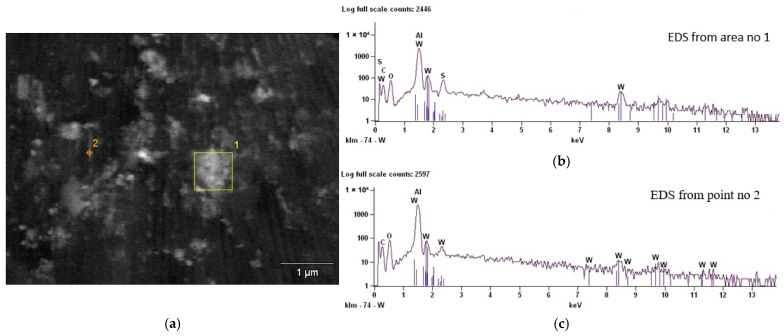
(**a**) SEM/YAG-BSE of a fresh fracture cross-section with a marked area of EDS analysis; (**b**) EDS from area 1; and (**c**) EDS from point 2 of Al_2_O_3_/IF-WS_2_ coating no. 2.

EDS analysis was conducted at the locations marked as area 1 and point 2 in Figure 2, summarising the results in Table 3. The “Atom%” column denotes the atomic percentage (at%) of each detected element, whereas the “Compnd%” column corresponds to the weight percentage (wt.%). The chemical composition of coating no. 2 was assessed by identifying and quantifying the elements that were present in the selected regions, with the results expressed as their respective compound formulas. The SEM/EDS cross-sectional analysis indicates that IF-WS_2_ nanoparticles can be introduced into the coating via the nanospaces between the alumina fibres. This structure may promote local retention of the additive, which can act as a nanolubricant reservoir in potentially tribologically active zones. It should be emphasised that the purpose of the EDS analysis was not to directly assess the uniformity of the additive distribution throughout the coating volume, but rather to confirm its presence and its ability to penetrate the porous structure. In this context, the effective accessibility of the IF-WS_2_ in the tribological contact zone and the potential for long-term interaction resulting from the presence of local nanolubricant reservoirs seem important. In both methods, the possibility of introducing IF-WS_2_ between the Al_2_O_3_ nanofibers was observed.

**Figure 3:**
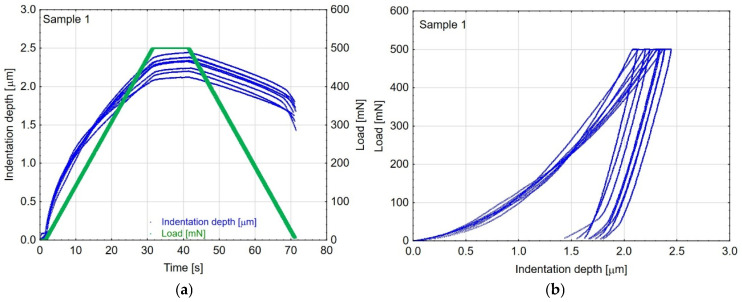
Examples of (**a**) indentation depth and load vs. time and (**b**) load vs. indentation depth, recorded during micromechanical tests on sample no. 1.

Microhardness tests were conducted on samples prepared using each fabrication method, with measurements repeated for every approach. A total of eight indentations were made on each sample. A representative graph illustrating the indentation depth and load as a function of time, along with the load–depth relationship, is presented in Figure 3. During the loading phase (0–35 s), with a linearly increasing load from 0 to 500 mN, the Vickers indenter’s penetration depth increases (Figure 3a), indicating material deformation. At the maximum load (35–40 s), the Vickers indenter reaches its most remarkable penetration depth. The maximum penetration depth for the exemplary sample number 1 ranges from 2.1 to 2.4 µm. As the load decreases to zero, the indentation depth also decreases but does not return to zero, indicating that the sample has undergone permanent deformation. A portion of the deformation is recovered (elasticity), while the remaining part persists (plasticity). The unloading curves (Figure 3b) exhibit material hysteresis, suggesting that the sample undergoes both elastic and plastic deformation. All tested samples exhibited the same material behaviour under the applied load described above.

**Figure 4:**
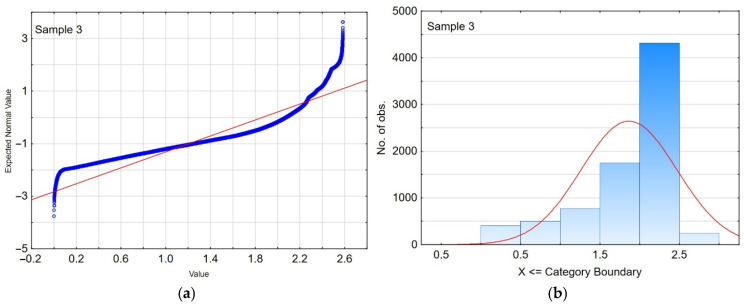
Example: (**a**) normality test and (**b**) histogram for sample no. 3.

The series of penetration depth curves obtained for each surface of the Al_2_O_3_/IF-WS_2_ coating was subjected to statistical analysis. The non-parametric Kruskal–Wallis ANOVA test was employed, since the measurement series for each sample did not follow a normal distribution (as exemplified for sample number 3 in Figure 4). This test is used to verify the hypothesis of no shift between the compared distributions, meaning that the differences between the medians of the examined variable across multiple populations are generally insignificant. However, it was assumed that the analysed distributions were similar. In each test, significant differences were found between the penetration depth measurements for each sample at the adopted significance level of α = 0.05. Nevertheless, a binary test analysis was conducted to further confirm the presence or absence of differences in the micromechanical parameters of the examined coatings.

**Figure 5:**
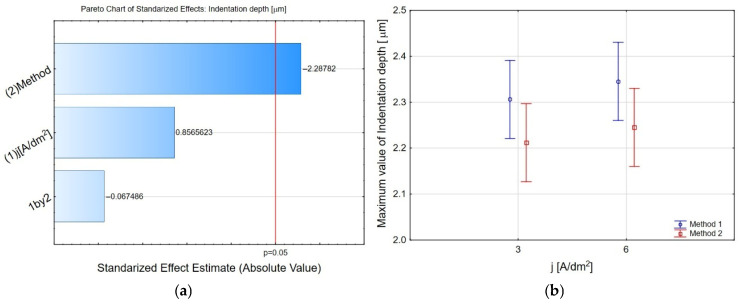
(**a**) The Pareto chart of standardised effects for the maximum value of indentation depth, and (**b**) charts of marginal means and confidence interval (95%), maximum value of indentation depth dependence on the method and electrolysis conditions.

To analyse the indentation depth parameter, the focus was on its maximum values. The Pareto chart shown in Figure 5a shows the significant effect of the coating modification method on the maximum indentation depth. Figure 5b shows the marginal average values for the maximum indentation depths as a function of the current density for coatings produced by both methods. For both current density values (3 and 6 A/dm^2^), samples obtained by method 2 exhibit lower maximum indentation depths compared to method 1. Increasing the current density from 3 to 6 A/dm^2^ results in a slight increase in the maximum indentation depth for both methods, suggesting a change in the coating microstructure (e.g., increased porosity or changes in matrix morphology) at higher j values. However, this trend is weaker for method 2, indicating greater stability of the mechanical properties of coatings obtained by this technique. It should be emphasised that the standard deviation ranges partially overlap, suggesting that the observed differences represent a trend, rather than a sharp boundary between the methods. However, the consistently lower indentation depths for method 2 across the tested current density range indicate a beneficial effect of this method on penetration resistance/lower deflection under load.

**Figure 6:**
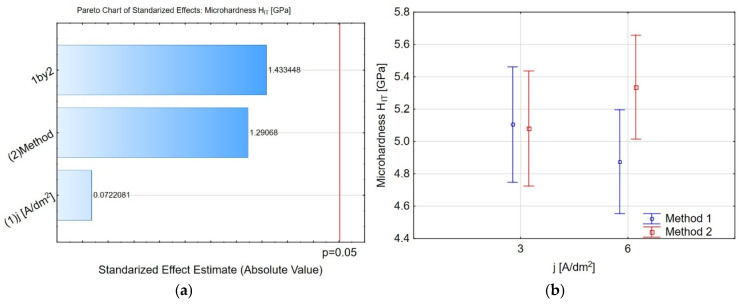
(**a**) The Pareto chart of standardised effects for the microhardness *H_IT_*, and (**b**) charts of marginal means and confidence interval (95%) for the microhardness *H_IT_* dependence on the method and electrolysis conditions.

**Figure 7:**
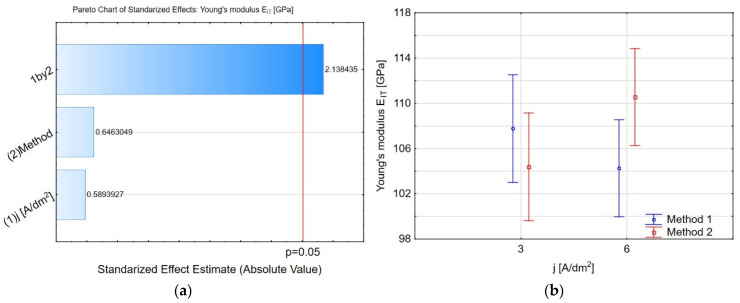
(**a**) The Pareto chart of standardised effect for the Young’s modulus *E_IT_*, and (**b**) charts of marginal means and confidence interval (95%) for the Young’s modulus *E_IT_* dependence on the method and electrolysis conditions.

**Figure 8:**
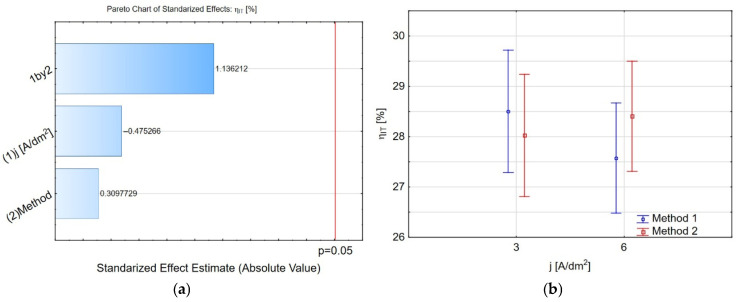
(**a**) The Pareto chart of standardised effect for the *η_IT_*, and (**b**) charts of marginal means and confidence interval (95%) for the *η_IT_* dependence on the method and electrolysis conditions.

**Figure 9:**
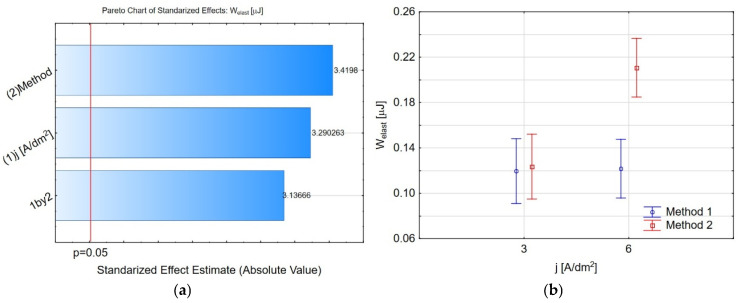
(**a**) The Pareto chart of standardised effect for the *W_elast_*, and (**b**) charts of marginal means and confidence interval (95%) for the *W_elast_* dependence on the method and electrolysis conditions.

**Figure 10:**
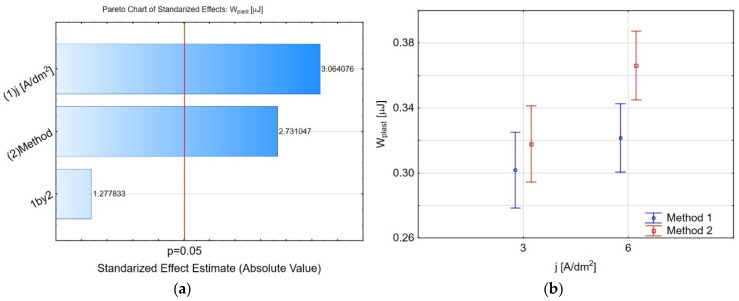
(**a**) The Pareto chart of standardised effect for the *W_plast_*, and (**b**) charts of marginal means and confidence interval (95%) for the *W_plast_* dependence on the method and electrolysis conditions.

Figure 6, Figure 7, Figure 8, Figure 9 and Figure 10 present selected Pareto charts, marginal mean plots, and confidence interval plots from the analysis of the two-level factorial design, respectively. Based on this analysis, the significance of coating fabrication parameters on micromechanical properties is discussed below. As shown in Figure 6a, neither the method of introducing IF-WS_2_ nanopowder into the Al_2_O_3_ coating nor the applied current density during the coating deposition significantly affects the microhardness of the examined coatings. For *j* = 3 A/dm^2^, the microhardness is similar for both methods: *H_IT_* = 5.10 ± 0.59 and *H_IT_* = 5.08 ± 0.62 for methods 1 and 2, respectively. For *j* = 6 A/dm^2^, the microhardness is slightly higher in the case of method 2 (*H_IT_* = 5.33 ± 0.65), but it does not differ significantly from method 1 (*H_IT_* = 4.87 ± 0.68). This observation aligns with previous studies [23], which showed that increasing the current density from 3.0 to 6.0 A/dm^2^ at a fixed anodising time did not significantly improve the average oxide microhardness. It is noteworthy that measurements were conducted at half the coating thickness. The authors of the cited work also reported that variations in current density at a constant process duration resulted in substantial changes in microhardness across the entire coating thickness. This relationship is associated with the varying thickness of aluminium oxide nanofibers across the coating’s cross-section and, consequently, with the coating’s surface porosity [21,22,46,47,49]. Conversely, the thickness of Al_2_O_3_ nanofibers is governed by chemical dissolution processes affecting the oxide surface and pore walls during exposure to the acidic electrolyte [50]. The slight increase in microhardness—still within the method 2 margin of error—observed in Figure 6b is consistent with the findings reported by the authors of the study [27], who demonstrated a rise in Vickers microhardness with the increasing current density. The length of the confidence intervals suggests some uncertainty in the results. However, the investigated methods for introducing IF-WS_2_ into Al_2_O_3_ coatings generally do not significantly alter their microhardness, which is more important from the perspective of the research presented here. The microhardness values of the investigated coatings range from 4.87 to 5.55 GPa, corresponding to 496–566 HV. Similar values were observed for coatings deposited on the EN AW-5754 alloy by Kessetini et al. [51], on the AlSi(Cu) alloy by Fratila et al. [23], on EN AW-1050 by Remesova et al. [20], and on EN AW-5052 by Dervishi et al. [10].

The Pareto charts (Figure 7a and Figure 8a) demonstrate that the interaction between the current density during coating deposition and introducing IF-WS_2_ nanopowder into Al_2_O_3_ is the only factor influencing the Young’s modulus value. For *j* = 3 A/dm^2^, the Young’s modulus values obtained using both methods are similar: *E_IT_* = 107 ± 11 and *E_IT_* = 104 ± 7, for methods 1 and 2, respectively (Figure 7b). For *j* = 6 A/dm^2^, method 2 yields a slightly higher value, *E_IT_* = 104 ± 7, than method 1, *E_IT_* = 110 ± 8. These values can be compared with the Young’s modulus values obtained for coatings produced in oxalic acid, as reported in [47], where the coating porosity was approximately 20%. The same behaviour is visible for the percentage of elastic recovery work *η_IT_* (Figure 8b). The methods of obtaining the Al_2_O_3_/IF-WS_2_ coating do not affect the *η_IT_* value.

The Pareto chart (Figure 9a) indicates that the method, current density, and their interaction significantly affect the elastic deformation energy *W_elast_*, as their bars exceed the significance threshold (*p* = 0.05). A similar trend is observed for the plastic deformation energy *W_plast_* (Figure 10a), except that the interaction between the manufacturing parameters does not influence it. At a higher current density (*j* = 6 A/dm^2^), higher values for *W_elast_* and *W_plast_* can be observed (Figure 9b and Figure 10b, respectively). These values are higher for method 2 of nanopowder incorporation into the coatings in both cases. Based on the obtained results, it can be concluded that for coatings produced at a current density of *j* = 6 A/dm^2^ using method 2 (application of nanopowder onto the coating), the highest amount of elastic deformation work (*W_elast_*) was accumulated during deformation. Simultaneously, this coating exhibited the highest value of plastic deformation work (*W_plast_*). Lower values of plastic deformation work *W_plast_* and elastic deformation work *W_elast_* are most likely due to the method of the incorporated IF-WS_2_ nanolubricant present on the coating surface and/or within its nanopores. Lower current density *j* = 3 A/dm^2^ and method 1 are suggested to be better for higher deformation resistance.

**Figure 11:**
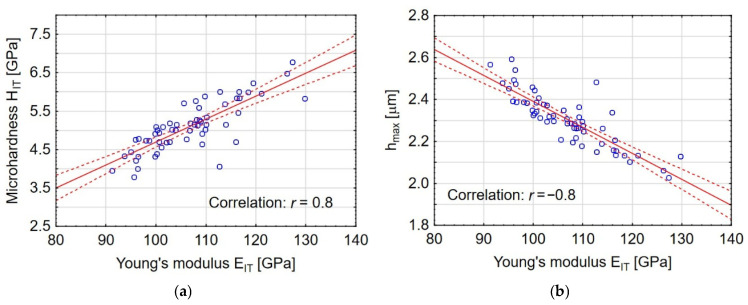

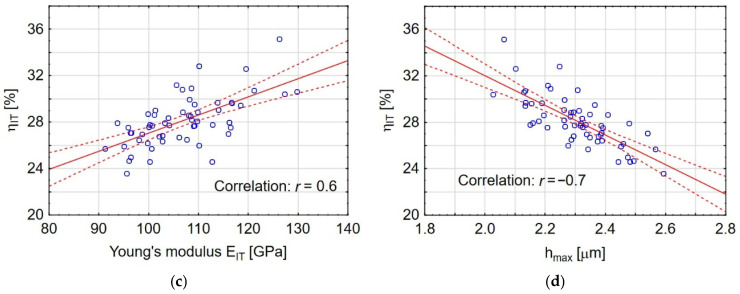
A scatter plot representing the relationship between (**a**) microhardness (*H_IT_*) and Young’s modulus (*E_IT_*); (**b**) maximum depth of indenter insertion (*h_max_*) and Young’s modulus (*E_IT_*); (**c**) share of elastic deformation work (*η_IT_*) and Young’s modulus (*E_IT_*); and (**d**) share of elastic deformation work (*η_IT_*) and maximum depth of indenter insertion (*h_max_*).

Figure 11 illustrates significant correlations between selected micromechanical property parameters. Figure 11a shows a scatter plot representing the relationship between microhardness *H_IT_* and Young’s modulus *E_IT_*. The plot includes a linear regression line, with dashed lines representing the confidence intervals. The correlation coefficient (*r*) is labelled as 0.8, indicating a strong positive correlation between the two variables. As the Young’s modulus increases, the microhardness tends to increase, reflecting a direct relationship between the two mechanical properties. A similar character of regression with a moderate correlation coefficient of *r* = 0.6 shows the relationship between the elastic recovery work *η_IT_* and the Young’s modulus (Figure 11c). In contrast, a high negative correlation of *r* = −0.8 has been shown for the maximum depth of indenter insertion *h_max_* and Young’s modulus (Figure 11b). A moderate correlation coefficient of *r* = −0.7 has been demonstrated for the elastic recovery work *η_IT_* and a maximum depth of indenter insertion *h_max_* (Figure 11d). The greater the penetration depth of the indenter, the less elastic energy is recovered from the coating. The indenter’s penetration depth mainly depends on the material’s porosity [47]. Our research also depends on the nanopowder introduced into the nanoporous microstructure.

By combining the micromechanical properties with the microstructural observations [35], some generalisations can be made. The observed effect of lower deflection under load for method 2 may be related to the greater number of IF-WS_2_ agglomerates accumulating in macropores on the coating surface, which promotes local strengthening of the contact zone during indentation. The presence of these agglomerates may limit direct indenter penetration into the Al_2_O_3_ matrix, resulting in lower maximum indentation depths despite no significant changes in microhardness. Unlike nanopores, which only promote the retention of single particles or small agglomerates, the macropores observed on the surface of coatings modified by method 2 allow for the accumulation of larger IF-WS_2_ agglomerates, which can act as local mechanical barriers, limiting indenter penetration and stabilising the mechanical response in the contact zone. At the same time, their presence promotes increased accumulation of elastic and plastic strain energy (*W_elast_* and *W_plast_*), indicating a more deformable yet stable mechanical response of the contact zone. The lower values for plastic (*W_plast_*) and elastic (*W_elast_*) strain work, observed for coatings obtained at a current density of *j* = 3 A/dm^2^ using method 1, most likely result from the presence of IF-WS_2_ nanopowder in smaller amounts on the coating surface and/or in its nanopores, where it acts as a nanolubricant, limiting strain development. In this context, a lower current density and method 1 may be advantageous in applications requiring limited accumulation of strain energy and increased resistance to permanent deformation, in contrast to method 2, which favours a more “working” mechanical response (the material deforms but does not collapse) associated with the presence of agglomerates in the macropores.

**Figure 12:**
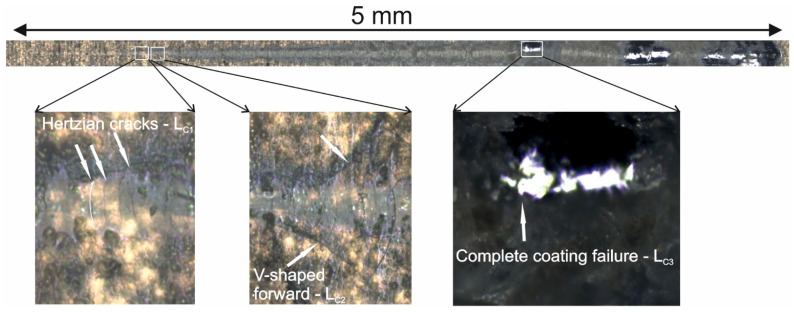
Scratch-track of sample 1.

Figure 12 shows a sample optical image of the scratch track for sample no. 1. The photos include representative graphs showing Hertzian cracks (*L_C__1_*), V-shaped forward (*L_C__2_*), and complete coating failure (*L_C__3_*).

**Figure 13:**
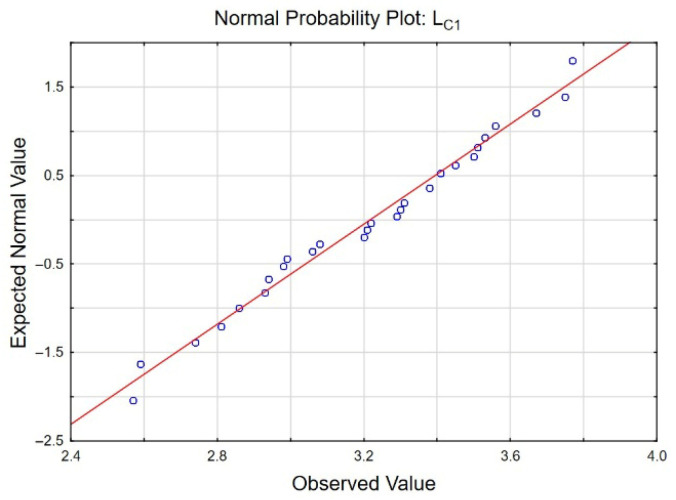
Normal probability plot for *L_C__1_*.

**Figure 14:**
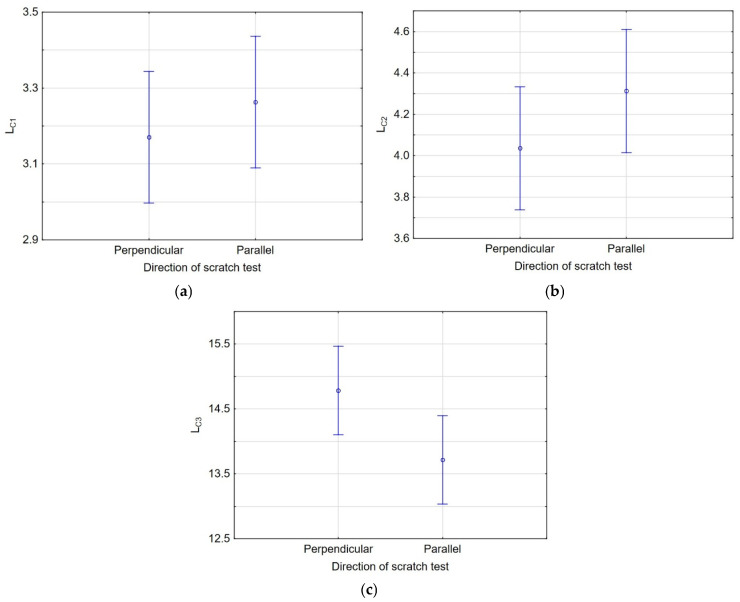
Expected mean values with a 95% confidence interval for (**a**) *L_C__1_*, (**b**) *L_C__2_*, and (**c**) *L_C__3_*.

All research results for *L_C__1_*, *L_C__2_*, and *L_C__3_* exhibited normal distributions (an example for *L_C__1_* is shown in Figure 13). Consequently, a one-way analysis of variance (ANOVA) was applied to assess the significant differences in measurements of *L_C__1_*, *L_C__2_*, and *L_C__3_* in two mutually perpendicular directions. The ANOVA results indicated a significant difference in *L_C__3_* measurements, indicating complete failure of the coating. In contrast, for *L_C__1_* and *L_C__2_*, the measurement direction of surface scratching was not a determining factor. Figure 14a–c present the expected mean values and 95% confidence intervals for *L_C__1_*, *L_C__2_*, and *L_C__3_*, respectively.

**Figure 15:**
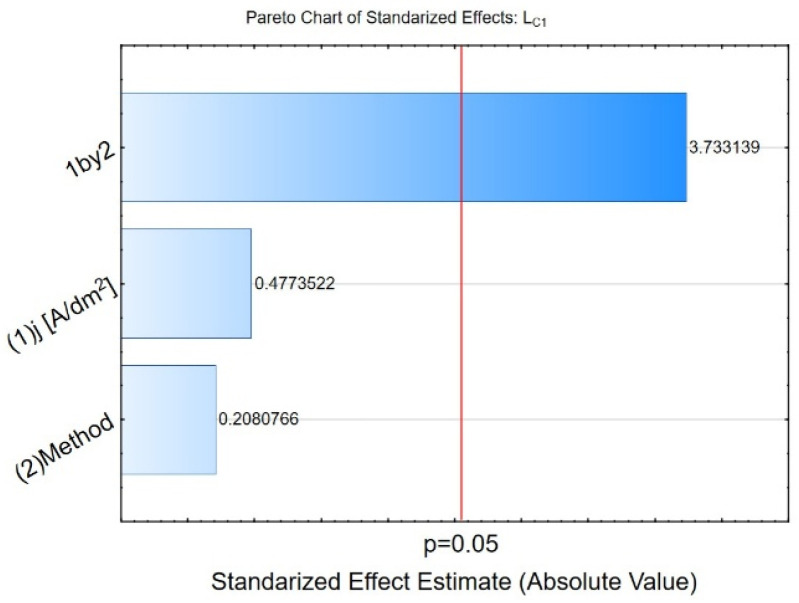
The Pareto chart of standardised effects for the *L_C__1_*.

**Figure 16:**
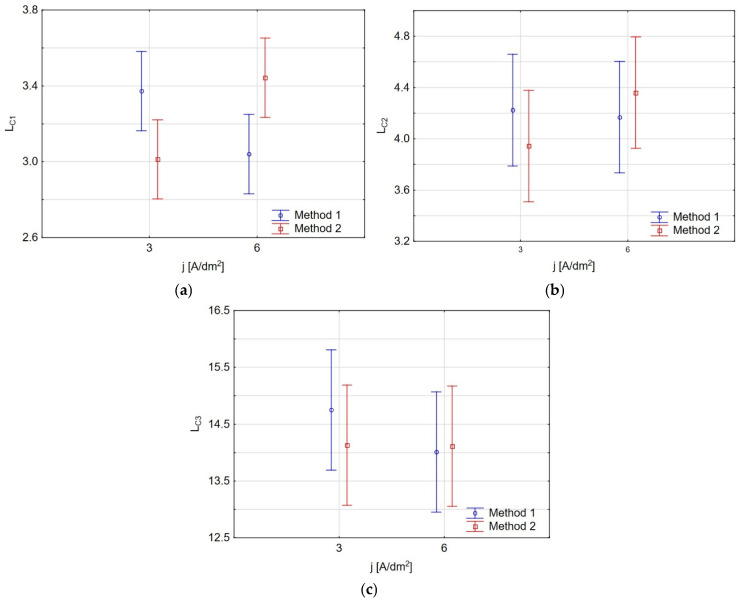
Charts of marginal means and confidence interval (95%) for the (**a**) *L_C__1_*, (**b**) *L_C__2_*, and (**c**) *L_C__3_* dependences on the method and electrolysis conditions.

A two-level factorial design analysis was performed for the critical load *L_C_* measurements. The results demonstrated a significant effect of the manufacturing parameters only on *L_C__1_* values, as shown in Figure 15. According to the graph, only the interaction (one by two) between the manufacturing parameters of the tested coatings significantly impacts the output value *L_C__1_*. Figure 16a–c show charts of marginal means and the confidence interval (95%) for the (a) *L_C__1_*, (b) *L_C__2_*, and (c) *L_C__3_* dependence on the method and electrolysis conditions. For *L_C1_* (Figure 16a), *j* = 3 A/dm^2^, method 1 shows a slightly higher (*L_C__1_* ≅ 3.35 N) value than method 2 (*L_C__1_* ≅ 3.01 N), but both values overlap within their error margins. For *j* = 6 A/dm^2^, method 2 exhibits a higher (*L_C__1_* ≅ 3.44 N) value than method 1 (*L_C__1_* ≅ 3.04 N), with a more noticeable separation. In the case of *L_C__2_* (Figure 16b) (at *j* = 3 A/dm^2^ *L_C__2_* ≅ 4.22 N (method 1); *L_C__2_* ≅ 3.94 N (method 2); at *j* = 6 A/dm^2^ *L_C__2_* ≅ 4.17 N (method 1); *L_C__2_* ≅ 4.36 N (method 2)), and *L_C__3_* (Figure 14c) at (at *j* = 3 A/dm^2^ *L_C__3_* ≅ 14.75 N (method 1); *L_C__3_* ≅ 14.13 N (method 2); at *j* = 6 A/dm^2^ *L_C__3_* ≅ 14.01 N (method 1); *L_C__3_* ≅ 14.11 N (method 2)), both methods exhibit similar *L_C__2_* and *L_C__3_* values, with overlapping error bars, indicating no significant difference between the two methods.

**Figure 17:**
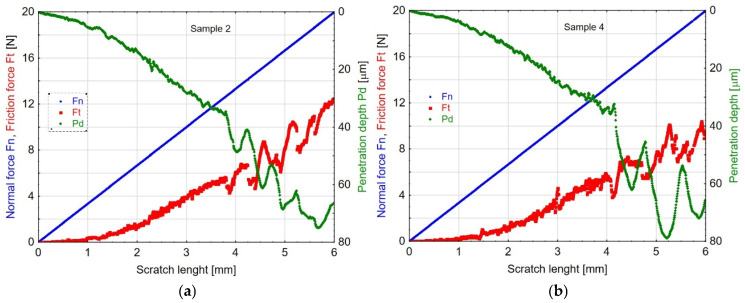
Characteristics of scratch test parameters, *Fn*—normal force, *Ft*—frictional force, and *Pd*—penetration depth, for samples 2 (**a**) and 4 (**b**).

The parameters recorded during the scratch tests of the coatings enabled the characterisation of friction force and indenter penetration depth under load (Figure 17). The *Fn* curve represents the normal force applied by the indenter, progressively increasing up to 20 N along the entire scratch length. Consequently, this characteristic remains identical for all tested coatings. In contrast, the *Ft* and *Pd* curves illustrate the variations in friction force and penetration depth as functions of scratch length. In all cases, the friction force of the indenter increases as it penetrates the coating and substrate structure—an expected outcome, due to the progressive increase in normal force.

**Figure 18:**
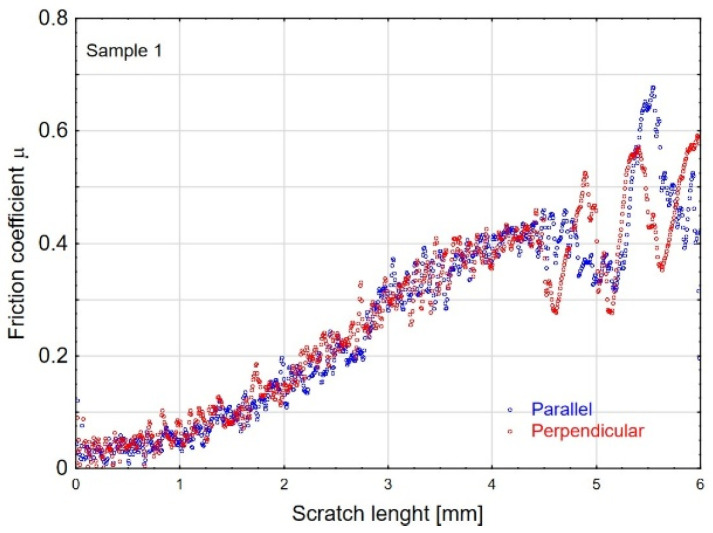
Friction coefficient vs. scratch lengths for sample no. 1.

In Figure 18, sample values (sample 1) of the friction coefficient are shown as a function of the distance travelled during the scratch test for scratches in two perpendicular directions. For the analysis of binary tests, it was assumed that the friction coefficient measurements are independent of the measurement direction.

**Figure 19:**
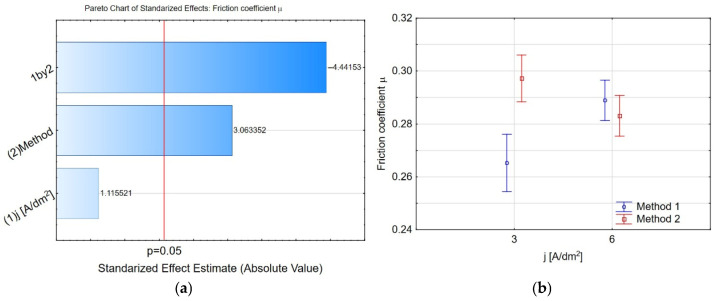
(**a**) The Pareto chart of standardised effects for the friction coefficient and (**b**) charts of marginal means and confidence interval (95%) for the friction coefficient dependence on the method and electrolysis conditions.

Figure 19a presents a Pareto chart, indicating that introducing nanopowder into the coating and the interaction between the preparation conditions (one by two) affect the friction coefficient. The provided graph (Figure 19b) illustrates the relationship between the friction coefficient *μ* and the current density *j* for methods 1 and 2. Each data point includes an error bar indicating the variability or uncertainty of the measurements. At *j* = 3 A/dm^2^, method 1 shows a lower friction coefficient with a more significant error margin than method 2. At *j* = 6 A/dm^2^, the friction coefficients for both methods are closer, with method 1 showing a slightly lower value than method 2. Overall, the graph suggests that the friction coefficient increases with the current density for method 1, whereas it remains relatively stable for method 2.

The stability of the friction coefficient observed for method 2 correlates with its ability to limit local penetration and with the presence of IF-WS_2_ nanoparticles, which act as local reservoirs that may favour a more controlled course of deformation and friction in the contact zone.

## 4. Conclusions

In this study, the current density and methods of incorporating nanoparticles into the microstructure have been applied to the aluminium alloy EN AW-5251 substrate, to produce porous anodic aluminium oxide coatings with the IF-WS_2_ nanolubricant. The effect of the above-mentioned condition on the resulting micromechanical properties of the porous Al_2_O_3_/IF-WS_2_ coatings was examined, and the following conclusion was drawn:

1. The IF-WS_2_ nanopowder introduction method and the deposition current density influence the mechanical and tribological response of Al_2_O_3_ coatings, with this effect manifesting primarily at the level of deformation and friction mechanisms, rather than in the form of significant changes in microhardness.
2. Method 2 leads to lower maximum indentation depths and greater stability of the mechanical response as a function of the current density, indicating improved resistance to local penetration.
3. At the same time, the lower current density (3 A/dm^2^) and method 1 help to reduce the accumulation of elastic and plastic strain energy, which may be beneficial in applications requiring increased resistance to permanent deformation.
4. DOE analysis confirmed that both the nanopowder introduction method and its interaction with the current density significantly affect the coefficient of friction, with method 2 demonstrating more stable tribological behaviour across the entire parameter range tested.
5. The obtained results indicate that there is no single, universally optimal technological variant, and the selection of the nanopowder introduction method and current density should depend on the dominant loading mechanism and the expected operational function of the coating.

## Figures and Tables

**Table 1:** The chemical composition of EN AW-5251 (wt.%).

Mn	Fe	Cu	Mg	Si	Zn	Cr	Ti	Other
0.1–0.50	≤0.50	≤0.15	1.70–2.40	≤0.40	≤0.15	≤0.15	≤0.15	≤0.15

**Table 2:** Conditions for coating formation and WS_2_ NPs incorporation, based on a two-level factorial design [35].

Sample Designation	The Electrolysis Conditions for Charge Density of 180 [A·min/dm^2^]		The Conditions of Introducing IF-WS_2_ NPs
	Time [min]	J [A/dm^2^]	
1	60	3	Method 1
2	60	3	Method 2
3	30	6	Method 1
4	30	6	Method 2

**Table 3:** Quantitative results for EDS from areas 1 and point 2.

Area	Element Line	Atom%	Atom% Error	Formula	Compnd%
1	O K	34.87	±1.81	O	21.19
	Al K	59.68	±0.45	Al	61.16
	S K	3.55	±0.24	S	4.32
	W L	1.91	±0.34	W	13.33
	Total	100.00			100.00
2	O K	34.79	±1.17	O	23.10
	Al K	64.61	±0.47	Al	72.33
	W L	0.60	±0.19	W	4.57
	Total	100.00			100.00

## Data Availability

The original contributions presented in this study are included in the article. Further inquiries can be directed to the corresponding author.
